# Retraction: MMP-2 siRNA Inhibits Radiation-Enhanced Invasiveness in Glioma Cells

**DOI:** 10.1371/journal.pone.0228689

**Published:** 2020-01-29

**Authors:** The PLOS ONE Editors

After this article [1] was published, concerns were raised about the following figures:

- In Fig 3, the U-251/p-SV and U-87/p-SV panels appear similar, as do the U-87/mock and U-87 mock/Irrad (8Gy) panels.
- In Fig 4, there appears to be overlap between the following panels:
  ○ U-251/mock (lower left) and U-251/mock/Irrad (8Gy) (upper right);
  ○ U-251/p-SV (left side) and U-251/mock/Irrad (8Gy) (right side);
  ○ U-251/p-MMP-2/2Gy (upper) and U-251/p-MMP-2/4Gy (lower).
- In Fig 5B, similarities were noted between bands in different lanes for each of the GAPDH panels.
- In Fig 6B, there appears to be overlap between the following panels:
  ○ [row 1] U-251/p-MMP-2/2Gy and U-251/p-MMP-2/4Gy;
  ○ [row 2] U-251/mock, U-251/p-MMP-2/6Gy, and U-251/p-MMP-2/8Gy;
  ○ [row 2] U-251/p-MMP-2/2Gy and U-251/p-MMP-2/4Gy;
  ○ [row 4] U-87/p-MMP-2/0Gy and U-87/p-MMP-2/8Gy.
- In Fig 6C, it is difficult to confirm the integrity of the blot images as the background appears uniformly white in most cases.

In light of the above concerns that call into question the integrity and reliability of the published results, the *PLOS ONE* Editors retract this article.

DA did not specify their position on this decision. The other authors either could not be reached or did not respond directly.

Note, the U-87 cell line from ATCC was reported in 2016 to be misidentified but likely of glioma origin [2].
